# Cytotoxicity of Cyanoacrylate-Based Tissue Adhesives and Short-Term Preclinical *In Vivo* Biocompatibility in Abdominal Hernia Repair

**DOI:** 10.1371/journal.pone.0157920

**Published:** 2016-06-20

**Authors:** Gemma Pascual, Sandra Sotomayor, Marta Rodríguez, Bárbara Pérez-Köhler, Andreé Kühnhardt, Mar Fernández-Gutiérrez, Julio San Román, Juan Manuel Bellón

**Affiliations:** 1 Department of Medicine and Medical Specialities, Faculty of Medicine and Health Sciences, University of Alcalá, Alcalá de Henares, Madrid, Spain; 2 Networking Biomedical Research Centre on Bioengineering, Biomaterials and Nanomedicine (CIBER-BBN), Madrid, Spain; 3 Department of Surgery, Medical and Social Sciences, Faculty of Medicine and Health Sciences, University of Alcalá, Alcalá de Henares, Madrid, Spain; 4 Biomaterials Group, Institute of Polymer Science and Technology (ICTP-CSIC), Madrid, Spain; University of Insubria, ITALY

## Abstract

**Background:**

Cyanoacrylate(CA)-based tissue adhesives, although not widely used, are a feasible option to fix a mesh during abdominal hernia repair, due to its fast action and great bond strength. Their main disadvantage, toxicity, can be mitigated by increasing the length of their alkyl chain. The objective was to assess the *in vitro* cytotoxicity and *in vivo* biocompatibility in hernia repair of CAs currently used in clinical practice (Glubran(n-butyl) and Ifabond(n-hexyl)) and a longer-chain CA (OCA(n-octyl)), that has never been used in the medical field.

**Methods:**

Formaldehyde release and cytotoxicity of unpolymerized(UCAs) and polymerized CAs(PCAs) were evaluated by macroscopic visual assessment, flow cytometry and Alamar Blue assays. In the preclinical evaluation, partial defects were created in the rabbit abdominal wall and repaired by fixing polypropylene prostheses using the CAs. At 14 days post-surgery, animals were euthanized for morphology, macrophage response and cell damage analyses.

**Results:**

Formaldehyde release was lower as the molecular weight of the monomer increased. The longest side-chain CA(OCA) showed the highest cytotoxicity in the UCA condition. However, after polymerization, was the one that showed better behavior on most occasions. *In vivo*, all CAs promoted optimal mesh fixation without displacements or detachments. Seroma was evident with the use of Glubran, (four of six animals: 4/6) and Ifabond (2/6), but it was reduced with the use of OCA (1/6). Significantly greater macrophage responses were observed in groups where Glubran and Ifabond were used vs. sutures and OCA. TUNEL-positive cells were significantly higher in the Glubran and OCA groups vs. the suture group.

**Conclusions:**

Although mild formaldehyde release occurred, OCA was the most cytotoxic during polymerization but the least once cured. The CAs promoted proper mesh fixation and have potential to replace traditional suturing techniques in hernia repair; the CAs exhibited good tissue integration and effective short-term biocompatibility, with the slightest seroma and macrophage response induced by OCA.

## Introduction

Most abdominal wall hernias are treated by the implantation of a prosthetic mesh in the damaged area. With the main objective of avoiding mesh displacement and thus the resulting recurrence, different fixing mechanisms have been used in open or laparoscopic repairs. Among the most widespread mesh-fixing mechanisms within the surgical scope are sutures and tacks; these are faced with surgical adhesives that are currently gaining popularity mainly because of their ability to reduce chronic pain and tissue damage in the patient [1]. It has been previously described that these deep mesh fixation devices might cause acute or chronic pain, increasing both direct and indirect health costs [2]. In fact, several research groups have proposed the use of surgical adhesives to secure mesh, which could lead to better results and avoid subsequent complications [3–5], with economic costs similar to those of conventional suturing.

Surgical adhesives, among others, include a group of synthetic glues called cyanoacrylates (CAs), which have been used in different medical applications due to their rapid action and great bond strength. The general chemical structure of the cyanoacrylate molecule is CH_2_ = C(CN)CO_2_R, with R representing an organic molecular group, e.g., ethyl (C_2_H_5_) or butyl (C_4_H_9_), in the variable alkyl group. “CA” is used to designate a set of these liquid monomers, cyanoacrylic esters, which polymerize exothermically in the presence of anionic components into long chains. These chains form a solid film with adhesive properties over an application surface [6].

Ideally, surgical adhesives should meet some essential requirements [7], such as biocompatibility with minimal tissue toxicity, adequate adhesive strength, polymerization in a moist environment, and gradual biodegradation without foreign-body/inflammatory response [8]. However, the use of commonly available CA adhesives presents two major problems. Tissue toxicity, which was initially related to the heat release of the exothermic reaction of polymerization [7] and subsequently attributed to the degradation of their alkyl chains into cyanoacetate and formaldehyde [9], prevents them from being metabolized and eliminated fast enough, resulting in tissue accumulation and inflammation [2]. Monomers showing short alkyl chains, such as methyl and ethyl, degrade faster than those with long chains, which implies a higher concentration of degradation products in the area of application, leading to cytotoxic and inflammatory effects and inhibiting the healing process; therefore, some have already been removed from medical use. The toxicity can be reduced with increased carbon numbers in the alkyl chains [10–12], which would slow degradation and therefore decrease the accumulation of toxic by-products [13, 14]. This fact has led to the development of new CAs, such as octyl CAs, and decreased recommendation of short-chain CAs for medical applications [2]. Materials for medical applications must be non-toxic and cause no harmful side effects. Therefore, the assessment of cell viability and cytotoxicity is a mandatory step in the evaluation of biocompatibility [15].

Flexibility is the other important problem associated with CAs because these polymers were typically hard and brittle after curing, which is undesirable for in vivo tissue conditions [7, 16]. Tissue injury due to CAs occurs in part because of the poor elasticity of the polymerized glue [17]. Improving the elastic properties of the CAs could therefore improve tissue reaction. Increased chain length is also related to increased polymer elasticity and decreased adhesive strength. Chain growth incorporating radicals produces more elastic adhesives, as increasing the number of chains, inter- and intramolecular forces are reduced [7].

In our study, one of the main toxic products released by the degradation of CA alkyl chains, formaldehyde, has been evaluated. Two different cell populations, fibroblasts (Fbs) and mesothelial cells (MCs), were used in *in vitro* studies to test the cytotoxicity of CA tissue adhesives. This study aimed for a more appropriate clinical translation because these two types of cells are the most likely candidates to contact with CAs in abdominal wall repair surgeries via an open surgical procedure or in intraabdominal placement of a prosthetic mesh during laparoscopic surgery, where TAs will be in contact with MCs covering the parietal peritoneum.

In this way, the present study was designed to assess the in vitro cytotoxicity exhibited by these two cell populations after exposure to CA tissue adhesives, which are currently used in clinical practice, with different alkyl chain lengths, Ifabond (n-hexyl) and Glubran 2 (n-butyl), in comparison with a new longer-chain CA adhesive (n-octyl), that has never been used in the medical field. Additionally, a short-term preclinical in vivo evaluation in a rabbit experimental model was also used to assess a specific application in the fixation of prosthetic meshes for the repair of abdominal wall defects. The *in vivo* performance of the different CAs was assessed in terms of biocompatibility and CA degradation.

## Materials and Methods

### Surgical adhesives

Cyanoacrylates for clinical use:

- Glubran 2: n-butyl CA (GEM S.R.L., Italy)
- Ifabond: n-hexyl CA (IFA Medical, France)
- Experimental n-octyl CA: (OCA); R = (CH_2_)_7_-CH_3_

### *In vitro* studies

#### Release of formaldehyde

Drops containing 30 μl of each cyanoacrylate adhesive were placed into sterile 10-ml polystyrene tubes. Monomers were immediately submerged in 6 ml of phosphate-buffered saline (PBS pH 7.4) and incubated at 37°C in a closed environment. At predetermined time points (0, 3, 6, and 24 hours and 2, 3, 4, 7, 10, 14, 17, 21, 28, 31, and 38 days), 300-μl aliquots of PBS were removed from each tube for further measurement of the formaldehyde concentration using a fluorometric detection kit, following the manufacturer’s instructions (Assay Designs, Ann Arbor, MI, USA). 50-μl aliquots from each sample, the standards provided with the kit and PBS (blank) were pipetted in duplicate into black 96-well plates. Then, 25 μl of the DetectX formaldehyde reagent were added to each well and the plates were incubated in darkness at 37°C for 30 minutes. Immediately following incubation, the fluorescent signal of each well was measured at 535 nm with excitation at 450 nm using a Victor2 microplate reader (PerkinElmer Wallac Inc., Finland). The results for each sample were averaged (n = 3) and plotted as the accumulative concentration of formaldehyde released by the different CAs over time.

#### Cell harvesting and culture

Dermis fibroblasts (Fbs) and mesothelial cells (MCs) were isolated from tissue biopsies of New Zealand white rabbits and collected at the Animal Research Center of Alcalá University. These animals were subsequently utilized in the preclinical study to adapt to the regulations of the three Rs.

- Fbs: Skin biopsies (approximately 1 cm^2^) were immersed in minimal essential medium (MEM) (Life Technologies Corporation, Carlsbad, CA, USA) and immediately processed under sterile conditions. The dermis was isolated with scalpel blades, diced into small explants, and subsequently cultured in 25-cm^2^ flasks using low-glucose Dulbecco's modified Eagle medium (DMEM) supplemented with 10% fetal bovine serum (FBS), penicillin-G 10,000 U/mL, streptomycin 10,000 μg/mL and amphotericin-B 25 μg/mL (Life Technologies Corporation). Culture flasks were kept in an incubator under a controlled humid atmosphere (37°C, 5% CO_2_) to allow the cells to migrate from the explant and colonize the flask surface. The media was changed every 3 days, and confluent cultures were treated with 0.25% trypsin-ethylenediaminetetraacetic acid (EDTA) at 37°C for 5 min, centrifuged at 200 g for 7 min, and transferred to culture flasks with 3 mL of complete DMEM at a 1:4 ratio. The cells were imaged using a Zeiss Axiovert 40C phase-contrast microscope (Carl Zeiss, Oberkochen, Germany). The cell cultures were exhaustively analyzed by light microscopy in order to verify that at least 90% of the population had the correct morphological characteristics, otherwise the cell cultures were discarded for the subsequent experiments.

- MCs: Omental biopsies (20–25 g) were obtained from the mesentery of the animals and processed under sterile conditions. The tissue samples were washed in MEM to remove the blood cells from the surface. Following this, the biopsies were immersed in a 0.1% collagenase type I dilution (Life Technologies Corporation) in MEM and were incubated at 37°C for 20 min under agitation (100 oscillations/min). Then, digested tissue was washed in 20 ml of MEM and discarded. The collagenase dilution containing the harvested MCs was mixed with the wash solution and centrifuged at 250 g for 10 min. After removing the supernatant, the cell precipitate was mixed in 10 ml of MEM and centrifuged again under the same conditions. Pelleted cells were resuspended in 4 ml of complete DMEM. The cell suspension was transferred to 25-cm^2^ polystyrene culture flasks and incubated under a controlled atmosphere, as previously described.

#### Cell interaction with the CAs

Under sterile conditions, the CA adhesives (10 μl each) were dropped in the center of wells in 6-well culture polystyrene plates. For each of the CAs tested, different groups were established based on the polymerization time:

- Unpolymerized cyanoacrylates (UCA)
- Polymerized cyanoacrylates for 1 day (1-PCA)
- Polymerized cyanoacrylates for 4 days (4-PCA)

The cells were exposed to adhesive glues by being seeded onto the different groups. The Cytotoxicity of the 3 CAs and cell viability of Fbs and MCs at the different time points (UCA, 1-PCA, 4-PCA) were evaluated by macroscopic visual assessment, flow cytometry and colorimetric Alamar Blue assays. The assays where carried out in triplicate and were individually developed for both Fbs and MCs.

#### Macroscopic visual assessment and cell viability

Aliquots of 3 x 10^5^ cells were seeded onto UCA, 1-PCA and 4-PCA wells (3 replicates per CA) and immediately filled with 2 ml of complete, phenol red-free DMEM. Cells grown in the absence of CAs were used as the control. The plates were incubated under a controlled atmosphere for 24 h. Macroscopic visual assessment of the cytotoxicity in the different cultures was performed. The cultured Fbs and MCs were imaged after 24 hours of exposure. Following incubation, the media was discarded and replaced with 2 ml of phenol red-free, FBS-free DMEM supplemented with 10% Alamar Blue reagent (AbD Serotec; Bio-Rad Laboratories Inc., Hercules, CA, USA). The plates were incubated for 5 h at 37°C, and medium from each well was collected (100 μl) to measure absorbance at 570 nm and 600 nm using an iMark microplate absorbance reader (Bio-Rad Laboratories Inc.). Wells containing medium with Alamar Blue but without cells served as a blank. The collected data were analyzed with an online colorimetric calculator provided by the manufacturer, available through the following link: http://www.abdserotec.com/colorimetric-calculator-fluorometric-alamarblue.html. The results were expressed as the mean percentage of Alamar Blue reduction of cells grown in the presence of cyanoacrylates versus control cells.

#### Flow cytometry and cell death

The confluent cultures were trypsinized, centrifuged and counted under a hemocytometer. Aliquots of 3 x 10^5^ cells were seeded onto UCA, 1-PCA and 4-PCA wells (3 replicates per CA) and immediately filled with 2 ml of complete DMEM. Cells grown in the absence of cyanoacrylates were used as a control. The plates were incubated under a controlled atmosphere for 24 h. Following this, the medium from each well was collected, and cells were harvested by trypsinization. The detached cells and culture medium were mixed, centrifuged (200 g for 7 min) and resuspended in 2 ml of flow cytometry staining buffer 1X (R&D Systems Inc., Minneapolis, MN, USA). The cell suspension was centrifuged again under the same conditions. Pelleted cells were resuspended in 400 μl of staining buffer and transferred to flow cytometry tubes, and 10 μl of propidium iodide (Sigma-Aldrich, St. Louis, MO, USA) was added to each tube. The rate of cell viability was determined using an argon laser (480 nm) FACSCalibur flow cytometer (BD Becton, Dickinson and Company, Durham, NC, USA), and data were analyzed using Cyflogic software (CyFlo. Ltd., Finland).

#### Statistical analysis

The results are expressed as the mean ± standard error of the mean (SEM). Data from the different groups were compared in pairs using the Mann-Whitney U test. All the statistical analyses were performed using the GraphPad Prism 5 computer package (GraphPad Software, Inc., La Jolla, CA, USA) for Windows. The significance level was set at p<0.05.

### *In vivo* preclinical studies

#### Experimental animals

The protocol of this study adheres to the ARRIVE guidelines for the publication of animal studies [18]. Eighteen male New Zealand white rabbits with a mean weight of 3000 g were used in this study. All research procedures and maintenance, handling, anesthesia and euthanasia of animals during the entire study period were performed in strict accordance with the recommendations detailed in the Guide for the Care and Use of Laboratory Animals of the National and European Institutes of Health (Spanish Law 6/2013, Spanish Royal Decree 53/2013 and European Convention of the Council of Europe ETS123). All procedures were performed at the Animal Research Center of the Alcalá University, which is registered with the Directorate General for Agriculture of the Ministry of Economy and Technology Innovation of the Community of Madrid (ES280050001165), indicating that all facilities legally cover the needs and requirements of the research. The Committee on the Ethics of Animal Experiments of the University of Alcalá approved the protocol of this study.

#### Prosthetic material

Polypropylene (PP) prostheses (Surgipro; Covidien, Mansfield, MA; USA) were used as the abdominal mesh for fixation with the different CAs.

#### Surgical technique

To minimize pain, all animals were administered 0.05 mg/kg of buprenorphine (Buprecare, Divasa Farmavic, Barcelona, Spain) 1 hour before and 3 days after the surgical procedure. Anesthesia was induced with a mixture of ketamine hydrochloride (70 mg/kg; Ketolar, Parke-Davis, Spain), diazepam (1.5 mg/kg; Valium, Roche, Spain), and chlorpromazine (1.5 mg/kg; Largactil, Rhone-Poulenc, Spain) administered intramuscularly. In some cases, an additional dose of anesthetic was injected directly in the abdominal cavity during the course of surgery.

Using a sterile surgical technique, defects (3x5 cm) were created in the anterior abdominal wall (right and left lateral side), comprising the external and internal oblique muscle and preserving the transverse muscle, transversalis fascia and parietal peritoneum. These defects were then repaired by securing the PP prostheses to the edges of the defect with 6 interrupted PP 4/0 sutures distributed in the four corners and in the upper and lower central area (control group) or with 6 drops (50 μl) of the different CAs (experimental groups). The skin was then closed over the implants with a running PP 3/0 suture. Throughout the study, the animals were visually inspected for signs of wound dehiscence, seroma formation, infection and/or areas of mesh incompatibility.

At 14 days after implantation, the animals were anesthetized with xylazine and were given a lethal dose of 20% sodium pentobarbital (Dolethal, Vetoquinol SA, France), as indicated by the procedures for the euthanasia of experimental animals.

#### Experimental design

A total of 18 animals were used. On the left side of some animals, the prosthesis (Surgipro, Covidien, USA) was implanted using interrupted sutures (control group) (n = 6). On the right side, the mesh was fixed with one of the CAs in a randomized way. The animals were distributed as follows:

- Glubran 2 (n = 6)
- Ifabond (n = 6)
- OCA (n = 6)

#### Morphological studies

Fragments of the implants including surrounding host tissue were obtained after euthanasia. Specimens were fixed in F-13 solution, embedded in paraffin, and sliced into 5-μm sections. Paraffin sections were deparaffinized in xylol and a graded alcohol series, hydrated, stained with Masson’s trichrome and examined using a light microscope (Carl Zeiss, Oberkochen, Germany).

#### Immunohistochemistry

To assess the macrophage response to the implants, macrophages were immunohistochemically detected in the paraffin-embedded sections of all the tissue samples from each animal included in the study using a monoclonal antibody to rabbit macrophages, RAM-11 (M-633, DAKO, Barcelona, Spain) and the alkaline phosphatase-labeled avidin-biotin method. Nuclei were counterstained for 5 minutes in acid hematoxylin. RAM-11-labeled macrophages were quantified by performing counts in 20 microscopic fields (200x magnification) for each sample in each group. Quantification was performed by 2 independent observers in a blinded fashion.

#### TUNEL

Cell damage was determined by a modification of the TUNEL (Terminal dUTP Nick End-Labeling) method [19]. A FragEL DNA fragmentation detection kit was used (QIA33, Calbiochem, CN Biosciences Inc., Boston, MA, USA). This technique is based on the in situ detection of cells with fragmented nucleosomal DNA by the specific addition of labeled nucleotides to the exposed 3′-OH ends of the chromatin fragments. The extent of binding is measured by assaying the enzyme terminal deoxynucleotidyl transferase (TdT). Positive cells were detected using a streptavidin-horseradish peroxidase (HRP) conjugate. Cell nuclei were counterstained with methyl green for characterization of non-damaged cells. TUNEL-positive cells were quantified in the same way as RAM-11-positive cells.

#### Statistical analysis

The results are shown as the mean ± standard error of the mean. The data for the different groups were compared in pairs using the Mann-Whitney U test. All statistical analyses were performed using the GraphPad Prism 5 computer package for Windows (GraphPad Software, Inc., La Jolla, CA, USA). Probability (p) values <0.05 were considered statistically significant.

## Results

### *In vitro* studies

#### Release of formaldehyde

**Fig 1:**
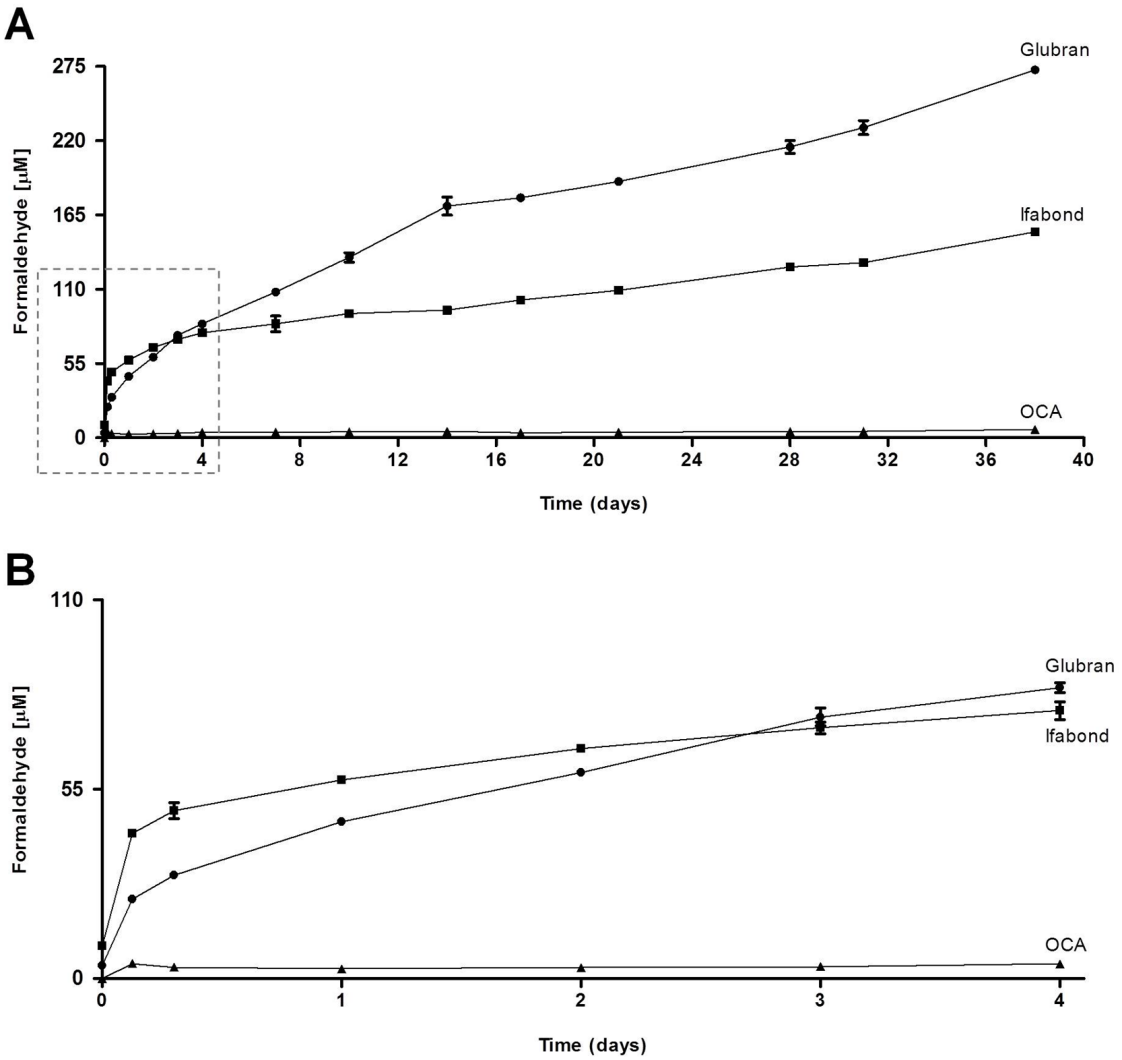
Release of formaldehyde. (A) Cumulative formaldehyde released [μM] by the different CAs used in the study (Glubran, Ifabond and OCA) after incubation in phosphate-buffered saline (PBS pH 7.4) at 37°C in a closed environment at the predetermined time points (0, 3, 6, and 24 hours and 2, 3, 4, 7, 10, 14, 17, 21, 28, 31, and 38 days). The test was performed using a fluorometric detection kit, following the manufacturer’s instructions (Assay Designs, Ann Arbor, MI). The results for each sample were averaged (n = 3). (B) Enlargement of the zone indicated with the dashed line box in A, for optimal analysis of the values of cumulative formaldehyde released [μM] by the different CAs in the initial rising phase before 4 days.

The release profiles of formaldehyde from the different CAs used in the study (Glubran, n-butyl; Ifabond, n-hexyl; and OCA, n-octyl) are shown in Fig 1. Generally, formaldehyde release decreased as the molecular weight of the monomer increased. The rate of release from OCA (n-octyl) was very low and remained so from the beginning to the end of the study period. Ifabond (n-hexyl) showed a higher release of formaldehyde than Glubran (n-butyl) did until approximately 3 days, after which the levels were reversed, increasing substantially in Glubran (n-butyl) until the end of the study period (Fig 1).

#### Macroscopic visual assessment

**Fig 2:**
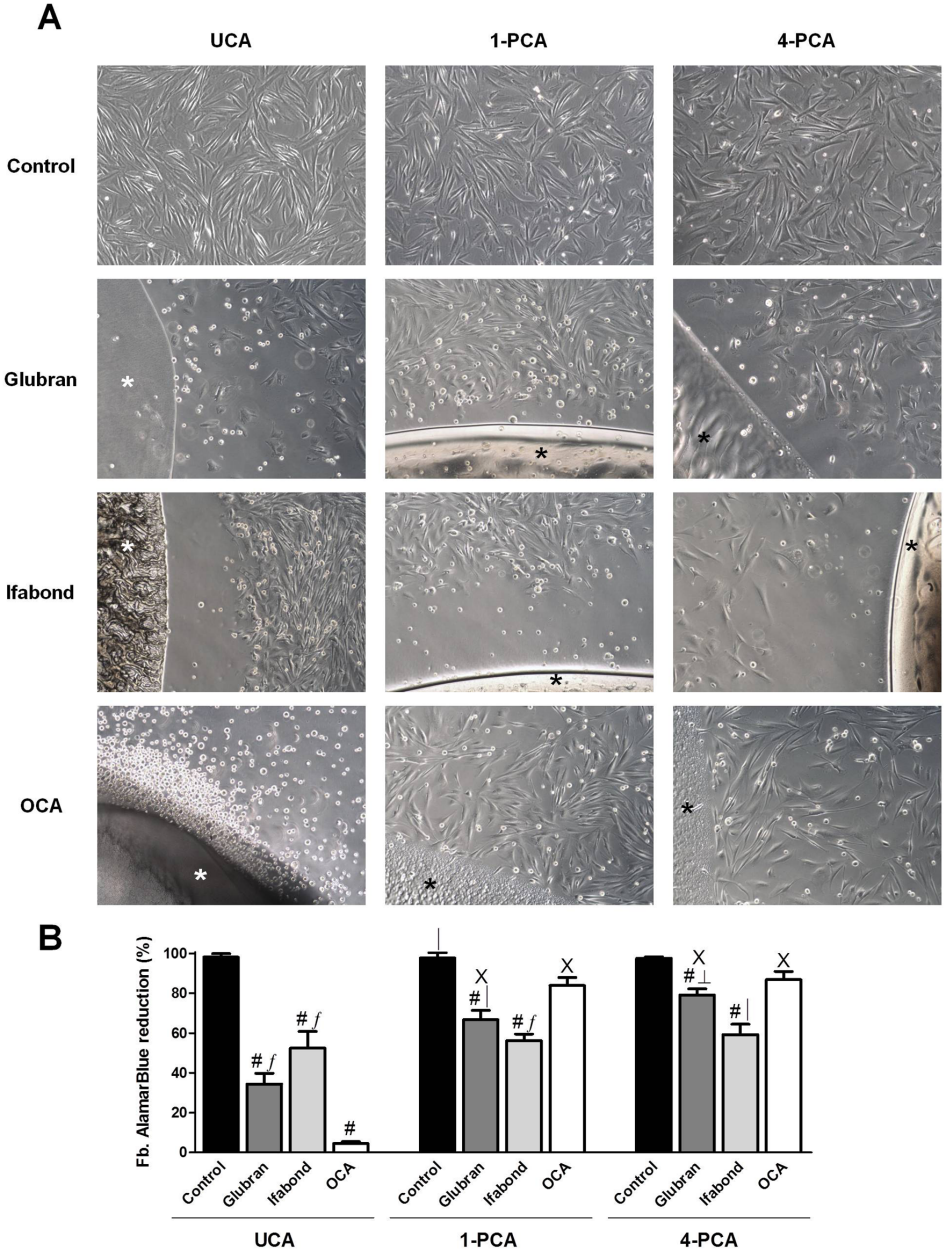
Fibroblast cell viability. (A) Macroscopic visual assessment. Images of the cultured fibroblasts after 24 hours of exposure to unpolymerized CAs (UCA) and polymerized for 1 and 4 days (1-PCA and 4-PCA) different CAs (Glubran, Ifabond, and OCA) vs. non-exposed cells (control) (100x). (*: Tissue Adhesive). (B) Mean percentage of the Alamar Blue reduction from fibroblast cultures after exposure to the different CAs. The results are expressed as the mean ± SEM for 3 independent assays performed in quadruplicate. Comparisons between groups: #: vs. control (***; p<0.001); ∫: vs. OCA (***; p<0.001); │: vs. OCA (**; p<0.01); ┴: vs Ifabond (**; p<0.01). Comparisons between times: Χ: vs. UCA (***; p<0.001).

-Fbs: In all cases, the control group showed a confluent cell monolayer with typical fibroblastoid morphology that covered the well (Fig 2A) at all study times.

UCA: The optical microscopy images showed that cells in the Glubran group (shorter alkyl chain) exhibited significantly cytotoxic macroscopic behavior characterized by the presence of few Fbs, showing morphological reversion to the spindle-shaped configuration, attached in the proximity of the areas with glue. A large percentage of the cell population exhibited mainly rounded cellular morphology, such as that of lysed cells. In Ifabond moderate cytotoxicity was observed, half of the population showed nonadherence and rounded morphology. However there was a big gap from the drop of glue to the area where fibroblasts were attached. The greater cytotoxicity was shown by OCA, most of the cells had rounded morphology of dead cells. (Fig 2A). In more remote areas, more adherent cells were observed, with large gaps between them.

1-PCA: Fbs in the Glubran and Ifabond groups presented similar macroscopic behavior when the CA drop was allowed to polymerize for 24 hours. Mild to moderate cytotoxicity was observed in both groups in areas close to the drop of glue, few adherent cells with altered fibroblastoid morphology were observed, and approximately a third of the population were lysed cells (Fig 2A). The big gap remained in Ifabond, without observing adherent cells near the glue. In more remote areas, cells that had adhered to the plate with small gaps between them could be observed. However, cells in the OCA group presented non-cytotoxic behavior, showing almost confluent Fb monolayer both in contact with the drop of glue and in remote areas (Fig 2A).

4-PCA: Moderate cytotoxicity was observed in the Ifabond group, with approximately a third of lysis occurring in areas close to the glue drop, as well as an altered fibroblastoid morphology in adhered cells and persistence of the gap. Glubran and OCA were non-cytotoxic, as adherent cells very close to the glue drops were observed in these groups. Fbs in the OCA group had attached nearer to the drop areas (Fig 2A).

**Fig 3:**
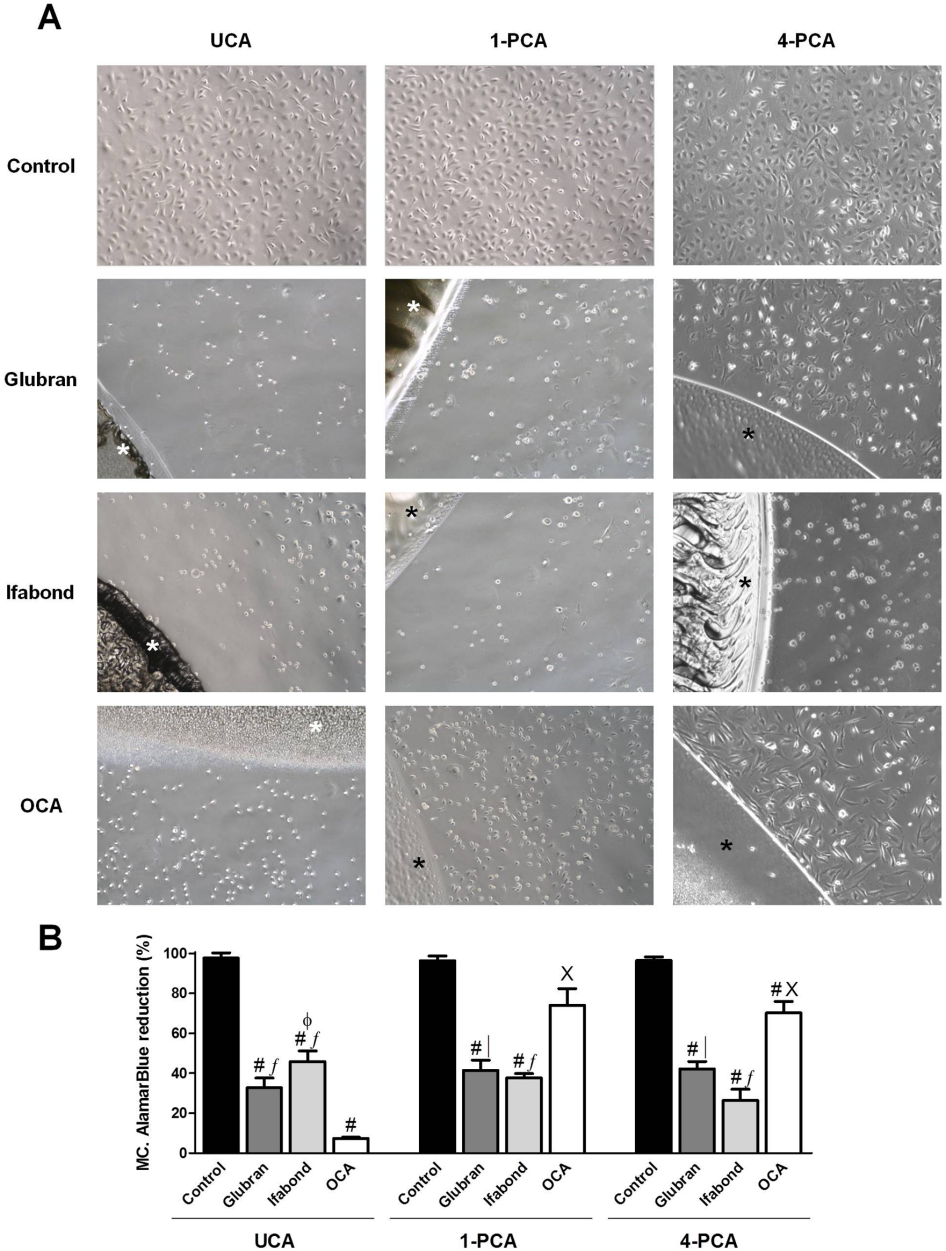
Mesothelial cell viability. (A) Macroscopic visual assessment. Images of the cultured mesothelial cells after 24 hours of exposure to unpolymerized CAs (UCA) and polymerized for 1 and 4 days (1-PCA and 4-PCA) different CAs (Glubran, Ifabond, and OCA) vs. non-exposed cells (control) (100x). (*: Tissue Adhesive). (B) Mean percentage of the Alamar Blue reduction from mesothelial cells treated with the different CAs. The results are expressed as the mean ± SEM for 3 independent assays performed in quadruplicate. Comparisons between groups: #: vs. control (***; p<0.001); ∫: vs. OCA (***; p<0.001); │: vs. OCA (**; p<0.01). Comparisons between times: X: vs. UCA (***; p<0.001); Φ: vs. 4-PCA (*; p<0.05).

-MCs: Images demonstrated that unexposed cells showed a typical polygonal monolayer culture exhibiting cell to cell contact at all study times (Fig 3A).

UCA: MCs showed increased susceptibility to cell damage that Fb. None of the three CA groups presented MCs attached adjacent to the drop of glue. OCA and Glubran showed significant cytotoxicity; most of the MCs were lysed and altered morphology was observed in areas remote from the drop. Ifabond showed moderate cytotoxicity showing lysis in half the population (Fig 3A), and cells with altered morphology in areas remote from the drop.

1-PCA: Adherent cells were observed closer to the bead of glue in the three CA groups, especially in the OCA group. Glubran and Ifabond showed moderate cytotoxicity, and OCA slight cytotoxicity with low cell lysis (Fig 3A). In remote areas, MCs were attached with small gaps between them in the three groups.

4-PCA: The cells exposed to Ifabond showed significant cytotoxicity, with a rounded morphology. Moderate cytotoxicity was observed in Glubrand. Most of the cells seeded over OCA appeared to be adhered near the margins of the adhesive; exerting no cytotoxic effects (Fig 3A).

#### Cell viability/Alamar Blue assay

- Fbs: As expected, the control group showed Alamar Blue reduction close to 100% (Fig 2B) in all cases and at all study times.

UCA: The results from the cell viability studies conducted with Alamar Blue showed that exposure to all the different CAs provoked a significant and acute decrease in the metabolic activity of the cultures versus the control group (p<0.001). OCA resulted in the lowest viability (4.48±0.89%), showing significant differences from the other two CAs (p<0.001) (Fig 2B).

1-PCA: One day after CA polymerization, increases viability were observed in all the CA groups; however, significant differences with respect to the control group were maintained. OCA resulted in the highest metabolic activity of all the CAs, showing significant differences from the other two CAs (p<0.01 vs. Glubran; p<0.001 vs. Ifabond) (Fig 2B).

4-PCA: The highest viability values were found at this study time. Significant differences with respect to the control group were maintained in the Glubran and Ifabond groups (p<0.001) but not in the OCA group, which resulted in viability close to 90%. Ifabond elicited a statistically significant decrease in metabolic activity compared with the rest of the CAs (p<0.01) (Fig 2B).

-MCs: As with the Fbs, nearly 100% viability was found in all the control groups (Fig 3B).

UCA: As with the Fbs, exposure to all the different CAs provoked a significant and acute decrease in the cellular metabolic activity compared with that of the control group (p<0.001). OCA resulted in the lowest viability (7.42±0.51%), which was significantly different from those of the Glubran and Ifabond groups (p<0.001) (Fig 3B).

1-PCA: After polymerization, OCA resulted in significantly increased viability values, reaching almost 75%; this result was significantly different from those of the other groups (p<0.001 vs. Ifabond; p<0.01 vs. Glubran), which maintained values similar to those observed in the UCA condition (Fig 3B).

4-PCA: Significant differences of all CAs with respect to the control were maintained (p<0.001). The metabolic activity observed after culture with OCA was similar to that observed in the 1-PCA condition, and the significant differences from the other CA groups were maintained (Fig 3B). The viability observed in the Ifabond group continued to decrease over time.

#### Flow cytometry/cell death

**Fig 4:**
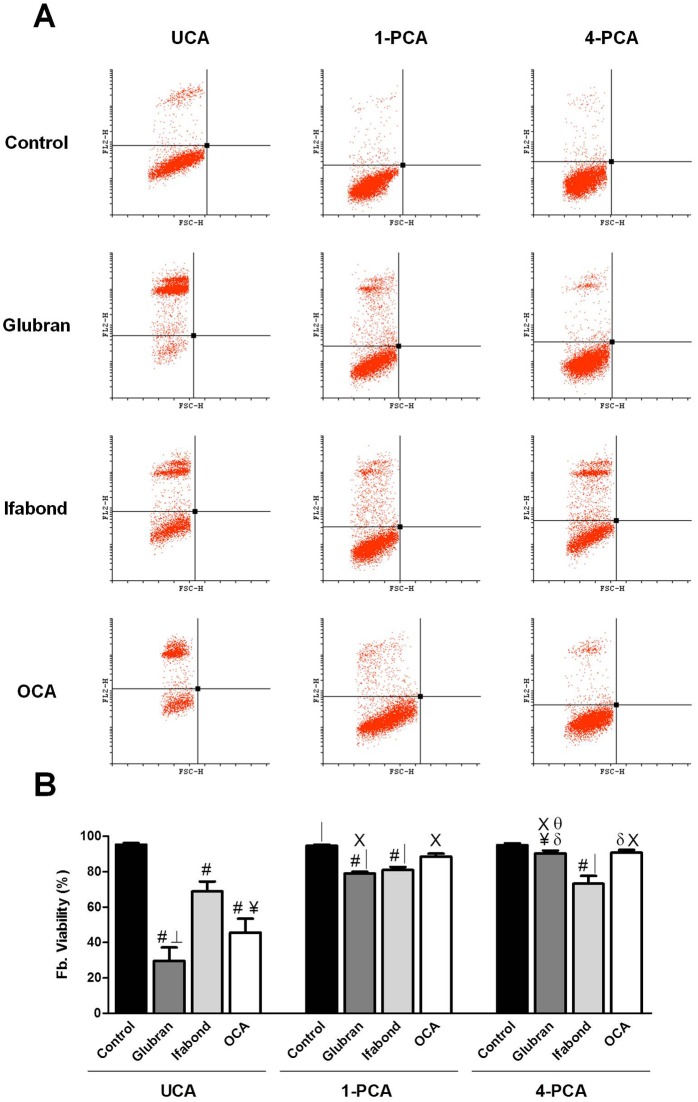
Fibroblast flow cytometry. (A) Scatter plots of the fibroblast cell populations after exposure to unpolymerized CAs (UCA) and polymerized for 1 and 4 days (1-PCA and 4-PCA) different CAs (Glubran, Ifabond, and OCA) vs. non-exposed cells (control) after 24 hours of culture. (B) Rate of cell viability of the fibroblast cell populations after exposure to the different CAs. The results are expressed as the mean ± SEM for 3 independent assays performed in quadruplicate. Comparisons between groups: #: vs. control (***; p<0.001); δ: vs. control (*; p<0.05); │: vs. OCA (**; p<0.01); ┴: vs Ifabond (**; p<0.01); ¥: vs Ifabond (*; p<0.05). Comparisons between times: Χ: vs. UCA (***; p<0.001); θ: vs. 1-PCA (***; p<0.001).

-Fbs: The control group showed a cell viability percentage close to 100% in all cases (Fig 4).

UCA: Quantitative studies of cell death showed significantly higher percentages of cell death in the three CA groups compared with that of the control (p <0.001). Ifabond resulted in a significantly higher number of viable cells compared with Glubran (p<0.01) and OCA (p<0.05). Cell viability after culture with OCA was greater than that of Glubran, but no significant differences between groups were found (Fig 4).

1-PCA: The three CA groups showed significantly lower cell viabilities than that of the control group (p <0.001, Glubran and Ifabond; p<0.01, OCA). In this condition, all CA groups showed higher values than they did in the UCA condition and over 75% viability. The OCA group showed a significantly higher percentage of live cells with respect to the Glubran and Ifabond (p<0.01) groups. No differences between the Glubran and Ifabond groups were observed (Fig 4).

4-PCA: The control group showed a significantly higher percentage of living cells than those of the three CA groups (p<0.001vs. Ifabond; p<0.05 vs. Glubran and OCA). The Glubran and OCA groups showed significantly higher viability percentages than that of the Ifabond group (p<0.05 and p<0.01, respectively). No differences between the Glubran and OCA groups were observed (Fig 4).

**Fig 5:**
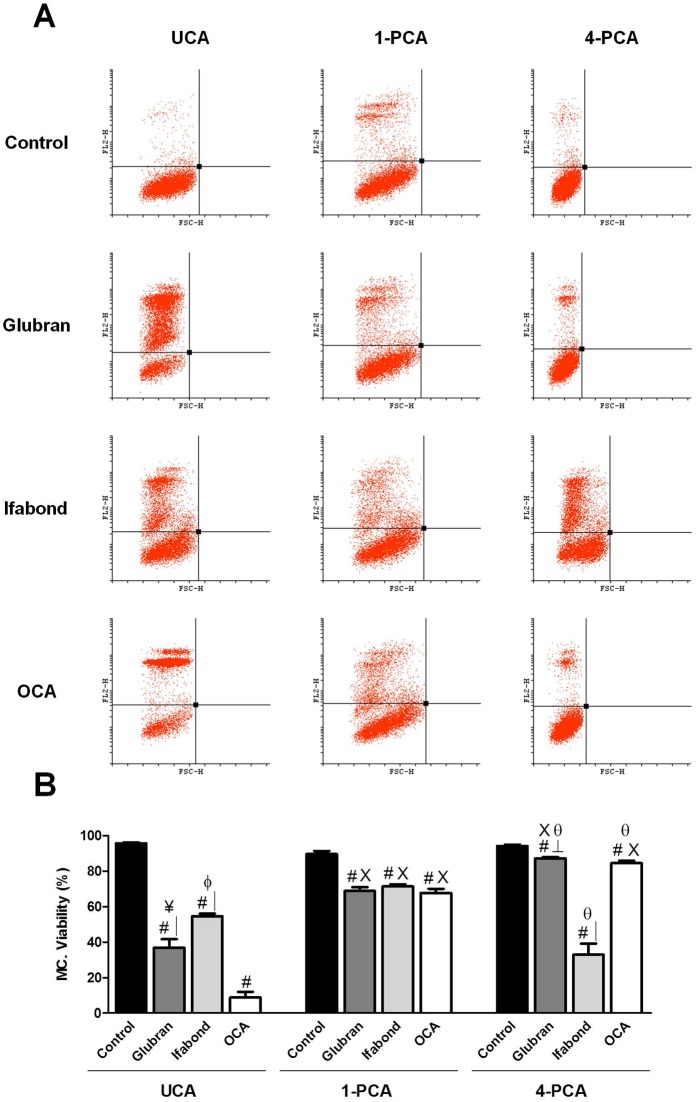
Mesothelial cell flow cytometry. (A) Scatter plots of the mesothelial cell populations after exposure to unpolymerized CAs (UCA) and polymerized for 1 and 4 days (1-PCA and 4-PCA) different CAs (Glubran, Ifabond, and OCA) vs. non-exposed cells (control) after 24 hours of culture. (B) Rate of cell viability of the mesothelial cell populations after exposure to the different CAs. The results are expressed as the mean ± SEM for 3 independent assays performed in quadruplicate. Comparisons between groups: #: vs. control (***; p<0.001); │: vs. OCA (***; p<0.001); ┴: vs. Ifabond (***; p<0.001); ¥: vs. Ifabond (**; p<0.01). Comparisons between times: X: vs. UCA (***; p<0.001); θ: vs. 1-PCA (***; p<0.001); Φ: vs. 4-PCA (*; p<0.05).

-MCs: As with the Fbs, nearly 100% cell viability was found in all the control groups (Fig 5).

UCA: The flow cytometry results showed that the three CA groups had significantly higher percentages of cell death than that of the control group (p<0.001). The Ifabond group showed a significantly higher percentage of live cells than those of the Glubran (p<0.01) and OCA (p <0.001) groups. Furthermore, the Glubran group showed a significantly higher viability percentage than that of the OCA group (p<0.001) (Fig 5).

1-PCA: The control group showed a significantly higher percentage of live cells than those of the three CA groups (p <0.001), showing greater values, close to 70% viability in all cases, than were observed in the UCA condition. No differences between the three CA groups were observed (Fig 5).

4-PCA: All the CA groups exhibited significantly lower cell viability percentages than that of the control group (p<0.001). The Ifabond group showed a significantly reduced viability compared with those of the other two CA groups, OCA and Glubran (p<0.001) (Fig 5).

### *In vivo* preclinical studies

#### Macroscopic analysis

All the different CAs and the sutures promoted optimal fixation of the prosthesis without causing displacements or detachments of the PP meshes. The presence of seroma was evident in the Glubran (four of six animals: 4/6) and Ifabond (2/6) groups. Seroma was also observed in some of the implants fixed with sutures (2/6). In the OCA group, seroma was reduced (1/6).

#### Morphological studies

**Fig 6:**
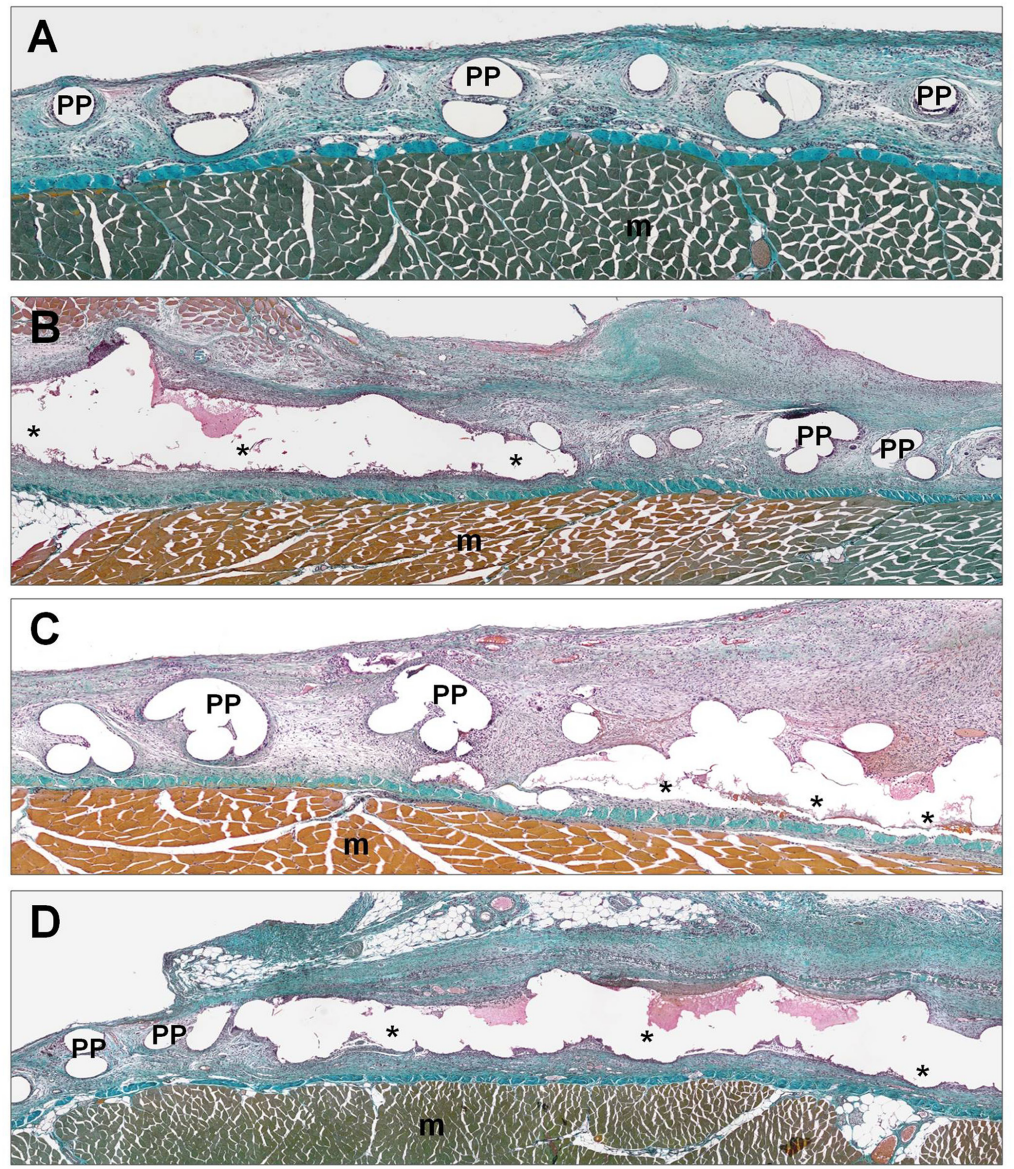
Morphological analysis. Panoramic light microscopy images of tissue integration of the reticular PP mesh (Surgipro) fixed with the different CAs (Glubran, Ifabond and OCA) and sutures (control group) 14 days after implantation. Masson’s trichrome staining (100x). (A) Image of PP mesh fixed with sutures in the control group (100x). (B) Image of PP mesh fixed with Glubran (100x). (C) Image of the PP mesh fixed with Ifabond (100x). (D) Image of PP mesh fixed with OCA (100x). (*: Tissue Adhesive; PP: polypropylene filaments; m: muscle).

In the control group, where the mesh was fixed with interrupted sutures, it was observed that the prosthesis was infiltrated by newly formed loose connective tissue surrounding the filaments and completely filling the space among the filaments of the mesh. A large number of small- and medium-sized blood vessels could also be observed throughout the area occupied by the interfilament, newly formed tissue. The behavior of the three CAs was similar. In the areas where the mesh was fixed with adhesive, seroma and the presence of fibrin were observed. In periprosthetic areas, the scar tissue was very cellular, with a loose fibrillar collagen network. At this study time, none of the different CAs used to fix the prosthesis were completely absorbed, and remnants of the CAs surrounded by foreign body giant cells and other inflammatory cells could be observed in all the different groups (Fig 6).

#### Macrophage response

**Fig 7:**
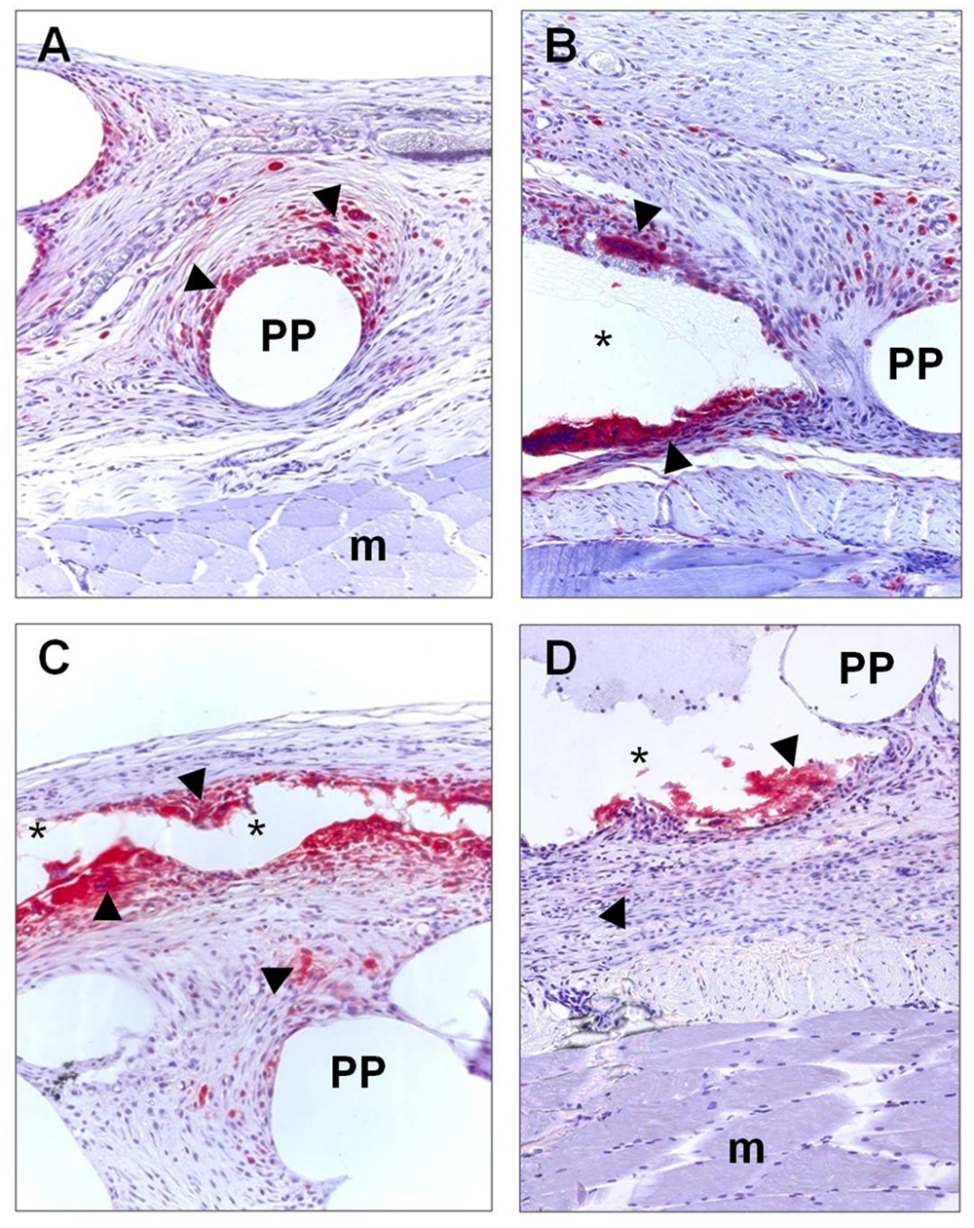
Macrophage response. Immunohistochemical labeling of rabbit macrophages (►) using RAM-11 monoclonal antibody in abdominal wall implanted with reticular PP mesh (Surgipro) fixed with sutures (control group) and with the different CAs (Glubran, Ifabond and OCA) 14 days after implantation. (A) Sutures, control group (200x). (B) Glubran (200x). (C) Ifabond (200x). (D) OCA. (200x). (*: Tissue Adhesive; PP: polypropylene filaments; m: muscle).

RAM-11-positive cells were significantly greater in the groups fixed with Ifabond and Glubran with respect to suture (control group) (p<0.001 in both comparisons) and OCA (p<0.01 in both comparisons) (Table 1). The macrophage response to OCA reached levels similar to that in response to suture. The macrophages and foreign body giant cells were present around the filaments of the mesh, and in the CA groups, they could be observed delimiting the area occupied by the adhesive bead. In some places, it was possible to see remnants of adhesive infiltrated in scar tissue surrounding the positive cells and foreign body giant cells (Fig 7).

**Table 1:** Percentage of RAM-11 and TUNEL-positive cells recorded for each study group.

	Data	Suture	Glubran	Ifabond	OCA
**RAM-11**					
	**Mean**	9.17	15.94	18.09	10.61
	**SEM**	± 0.84	± 1.33	± 1.56	± 1.37
	**Probability (p)**		# │	# │	
**TUNEL**					
	**Mean**	19.28	35.87	24.42	34.05
	**SEM**	± 1.77	± 4.03	± 2.58	± 5.68
	**Probability (p)**		# ¥		δ

^#^: (***; p<0.001) vs. Suture;

^│^: (**; p<0.01) vs. OCA;

^δ^: (*; p<0.05) vs. Suture;

^¥^: (*; p<0.05) vs Ifabond.

#### TUNEL

**Fig 8:**
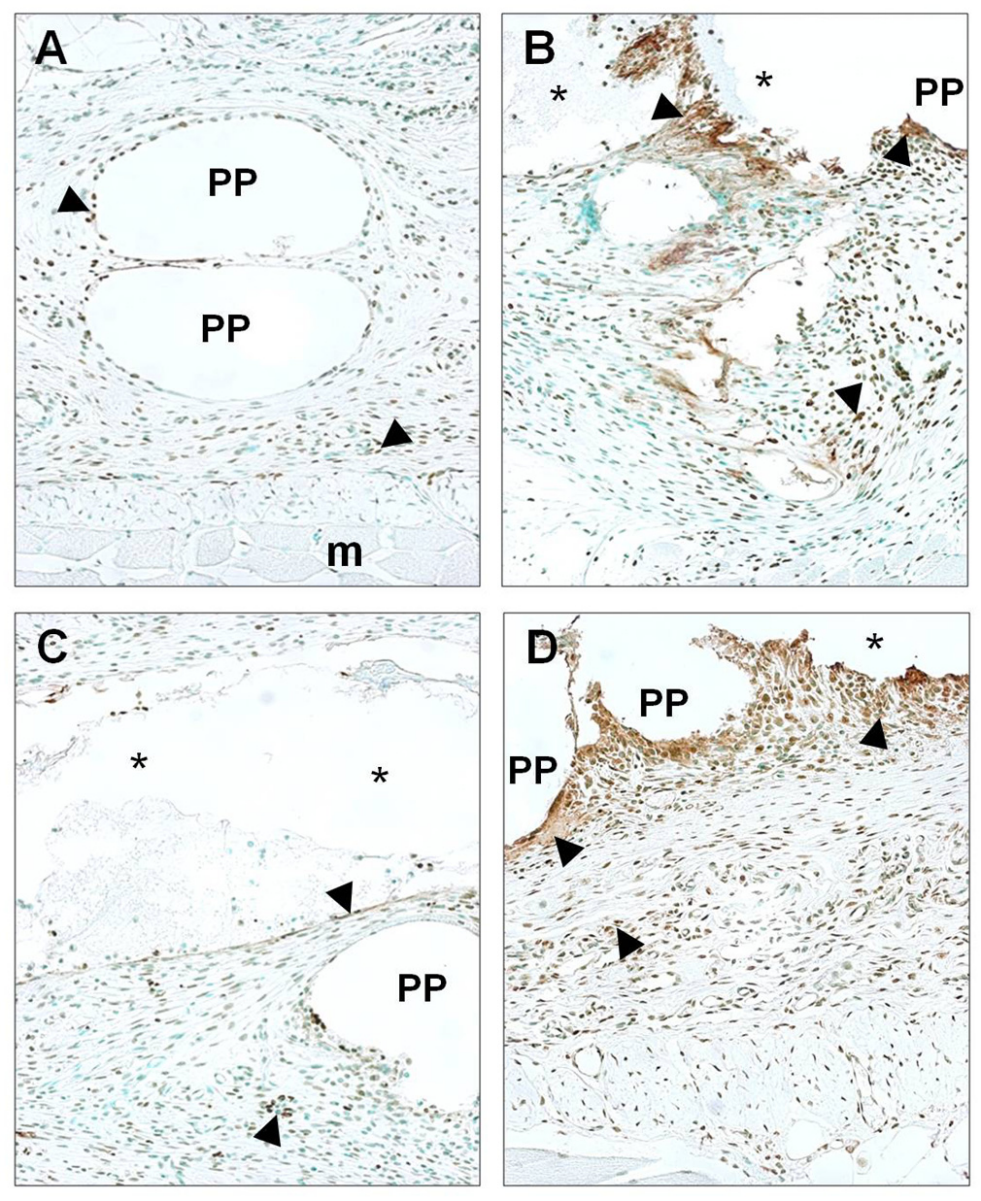
Cell damage. TUNEL-positive cells (►) in abdominal wall implanted with reticular PP mesh (Surgipro) fixed with sutures (control group) and with the different CAs (Glubran, Ifabond and OCA) 14 days after implantation. (A) Sutures, control group (200x). (B) Glubran (200x). (C) Ifabond (200x). (D) OCA (200x). (*: Tissue Adhesive; PP: polypropylene filaments; m: muscle).

In general, the positive cells were distributed throughout the neoformed tissue. In the suture group, the labeled cells were mainly located around the PP mesh filaments. In the CA groups, the labeled cells were located further along the CA drops and deeper in the newly formed tissue of these areas (Fig 8).

After two weeks of implantation, the percentage of TUNEL-positive cells was higher in the Glubran and OCA groups than in the suture group (p<0.001 and p<0.05, respectively). Significant differences between the Glubran and Ifabond groups (p<0.05) were also observed (Table 1).

## Discussion

One of the most serious problems of the surgical use of CAs involves its degradation and toxicity. In the in vitro part of our study, we assessed the cytotoxicity of different CA tissue adhesives, which are currently used in clinical practice, with different alkyl chain lengths, Ifabond (n-hexyl) and Glubran (n-butyl), in comparison with the new longer-chain CA adhesive, OCA (n-octyl). These products degrade and form cyanoacetate and formaldehyde, the latter of which is the main cause of CA toxicity; therefore, it was the first candidate evaluated in our in vitro study. Some evidence has already been shown that formaldehyde may be toxic to cells in vitro [20, 21]. In our study, we observed that formaldehyde release decreased as the molecular weight of the monomer increased. The rate of release from the n-octyl adhesive was very low and remained so from the beginning to the end of the study period; however, the n-hexyl and n-butyl adhesives exhibited higher formaldehyde release levels. The measurements recorded in our study with this protocol correspond to the concentration of formaldehyde being released by the different CAs over time. The highest levels were observed for the n-butyl adhesive, which were still increasing linearly after 38 days.

Our results are in accordance with those of other reports [7] that have shown that monomers with longer side-chains release less formaldehyde. However, these authors allowed the CAs to polymerize for 24 hours at room temperature, what can lead to the loss of formaldehyde released before those 24 hours, and in our study, the monomers were immediately submerged in the phosphate-buffered saline without prepolymerization.

In vitro, visual cytotoxicity scoring method has been developed based on the observable characteristics of cell spreading and cell lysis of mouse Fb in contact with different biomaterials, including tissue adhesives as representative biomaterials intended for use in the abdominal space [22]. This has been correlated with quantitative cell viability measurement methods, such as the MTT colorimetric assay. In our study, in vitro cytotoxicity was assessed using two different cell populations (i.e., mesothelial cells and fibroblasts) with which the CAs would be susceptible to contact in intra- or extraperitoneal abdominal hernia repair. In addition, the cells in our study were visually examined and characterized by flow cytometry and an Alamar Blue assay. Different groups were performed to evaluate adhesive cytotoxicity during and after polymerization of the CAs. Surprisingly, when the cells were exposed to uncured CAs, OCA resulted in a higher percentage of cell death and reduced viability regardless of cell type and study technique used, despite being the CA with the longest side-chain and lowest formaldehyde release. However, 1 and 4 days after polymerization (1-PCA and 4-PCA), the results were reversed for this group, and it resulted in higher viability on most occasions. This increased early cytotoxicity of the n-octyl adhesive could be due to the action of other released toxic compounds or the failure of local heat dissipation from the exothermic polymerization reaction, which can generate significant local increases in temperature and, consequently, cell death.

Other authors [7] who have worked with commercial n-octyl adhesives, such as Dermabond, have also reported greater cytotoxicity of these CAs compared with that of alkoxyethyl and hexoxyethyl CAs, even though there was less formaldehyde released. They attributed this toxicity to other products [23], such as alkyl alcohols, released by hydrolysis of the side-chains, which are more likely to be cytotoxic than alkoxyethyl alcohols. However, these results are not truly consistent with ours because these authors seeded the cells over glues that had been cured for 24 hours, and under these conditions, we observed that the n-octyl adhesive was the least toxic CA, showing very high cell viabilities in all cases.

With extensive experience in studying the cytotoxicity of a variety of materials, the research group of Montanaro [8] has established a reasonable threshold for cell viability to identify toxicity; toxicity is indicated if a viability rate is <70%, and non-toxicity is indicated by a viability >70%. In this work, which used n-ethyl and n-butyl CAs, severe cytotoxicity of both undiluted adhesives were observed; nevertheless, after a 1:10 dilution, fibroblast cytotoxicity was acceptable, with more than 70% viable cells. According to this threshold, our Alamar Blue cell viability results, which were the most restrictive, showed no toxicity in both cell populations only when exposed to prepolymerized OCA and in Fbs when exposed to Glubran in the 4-PCA condition. All CAs resulted in cell viabilities lower than 70% and were therefore toxic, in the UCA condition. Our flow cytometry results were not as restrictive, showing percentages of cell viability greater than 70% in most CAs in 1PCA and 4-PCA conditions. In contrast, Kukleta et al. [1], as one of the first surgeons to routinely use tissue adhesives for fixing meshes in abdominal repairs, has shown low toxicity of n-butyl CA extracts on L929 mouse fibroblasts.

Other groups [15] have assessed the in vitro cytotoxicity of new PACA (partial pre-polymerization allyl 2-CA) tissue adhesives and commercially available n-octyl CAs, such as Dermabond. These authors have previously shown that PACA causes longer chain structures that led to improved biocompatibility of common CAs [24] and observed that direct contact resulted in the lowest cell viability at 24 hours, close to 50%, and no cytotoxicity was observed using the indirect method.

The use of CAs in clinical practice is well described in the literature [25] and has become a standard tool frequently used for the closure of skin wounds and small lacerations of the body. This noninvasive adhesive fixation method is currently becoming increasingly popular in herniorrhaphy to prevent mesh dislocation and reduce the risks of damage, percutaneous injuries from other anchoring devices, such as suture needles, and postoperative complications, such as chronic pain [1]. Numerous in vitro animal studies have confirmed the safety of chemical and biological adhesives for mesh fixation [26, 27]. As an experimental model validated by our group, the New Zealand white rabbit has provided excellent results in terms of tissue repair and immune response. However, this study is not without its limitations, and the results from our model are difficult to translate to human clinical practice. Clinical studies have shown that chemical adhesives are an effective means of mesh fixation in hernia repair, and the results have been comparable to those of traditional techniques, such as suture and tack devices [28]. A fairly complete review [29] has analyzed trials comparing suture mesh fixation versus glue mesh fixation in open inguinal hernia repair, reporting that both are comparable in terms of postoperative complications, postoperative pain, chronic groin pain and length of hospital stay. Recent studies [30, 31] using n-butyl-2-cyanoacrylate for mesh fixation in hernia repair have showed that this chemical adhesive provides satisfactory performance for patch fixation in inguinal herniorrhaphy, did not result in an increased rate of hernia recurrence and provided reduced postoperative pain, reduced hematoma formation, and a lower incidence of chronic pain with respect to suture fixation.

In vivo tissue toxicity of CAs has been suggested to occur in different ways that affect compound biocompatibility. Foreign body reaction is the first thing to take into account and is provoked by a low absorption of the CA that leads to macrophage infiltration as well as polymorphonuclear cells attempting to remove the polymerized CA and the rest of the damaged tissue. This process, along with the production and accumulation of formaldehyde and cyanoacetate, which cause cell death and release of oxygen free radicals, contributes to the loss of tissue and promotes the release of different mediators, which exacerbate local ischemia, necrosis and tissue damage processes [2]. It has also been suggested that tissue injury due to CAs occurs in part because of the poor elasticity of the polymerized glue [17]. For this reason, some groups have been working on the incorporation of an etheric oxygen side-chain in place of an alkyl group, improving the elastic properties of hexoxyethyl CA over its commercially available and widely used alkyl analog 2-octyl CA without compromising biocompatibility [7].

Both aspects, foreign body reaction and tissue damage, have been evaluated in our preclinical study. Significantly greater macrophage responses were observed in groups fixed with CAs Glubran (n-butyl) and Ifabond (n-hexyl) than in the suture group (control) and in the group of implants fixed with OCA (n-octyl). The presence of macrophages and foreign body giant cells was observed delimiting the area occupied by the adhesive bead, and sometimes in scar tissue, but did not compromise the healing process. Other papers [32,33] have speculated about the occurrence of a foreign body reaction after the use of 2-octyl cyanoacrylate, but no histopathological analysis supporting their theory has yet been published. Fortelny et al. [34], in a model of chronic hernia in the midline of the rat abdominal wall, used Glubran 2 (butyl-2-CA) and found that tissue integration of the meshes was impaired by unresorbed rigid glue residues; significant inflammation appeared in the area, which would affect the healing of the surgical wound. In summary, they stated that this butyl-2-CA is not suitable for ventral hernia repair with macroporous meshes, in contrast with the results of other groups, including ours. In our case, optimal tissue integration was observed 14 days after implantation, showing physiological inflammatory reaction. A problem of this study might be that the amount of adhesive used was excessive, producing a severe inflammatory reaction and interfering in the physiological process of healing.

Different studies comparing CA adhesives and the standard suture for patch fixation in Lichtenstein herniorrhaphy have concluded that there are no great differences in the progress of healing or in the safety of the procedures and that CAs can be considered viable options [30, 35]. Other results support the use of CAs for the fixation of abdominal meshes [27, 28] and have shown in experimental models by measuring parameters, such as number of adhesions, degree of inflammation and presence of infection, that the adhesives do not show significant differences compared with standard suturing.

With respect to in vivo cell damage, the percentage of TUNEL-positive cells in our study was significantly higher after two weeks in the Glubran and OCA groups compared with the suture control group. These results correlated with the in vitro results of cell exposure to the UCA condition, where the OCA and Glubran groups also exhibited higher cell death and reduced viability. As already mentioned, this could be due to the exothermicity of the polymerization reaction or to the action of toxic compounds released by the glue other than formaldehyde leading to cell death. Other authors [28] using experimental models of partial defects of abdominal wall in the rat, in contrast with our study, have shown an absence of necrosis and apoptotic cells in tissue areas near the area of prosthesis fixation with n-butyl-2-CA. They have also described, in accordance with our results, a mild inflammatory reaction at CA application sites, with a small number of macrophages and the presence vascularized connective tissue around the glue and mesh threads as well as some CA residues observed in the tissue sections.

Comparative studies [36] using sutures, n-butyl-2-CA and human fibrin glue for mesh fixation during primary inguinal hernia repair have also shown no relapse and superior performance with fewer foreign body reactions by both types of adhesives with respect to traditional sutures. The clinical and experimental data of Kukleta et al. [1] also supported the effectiveness of CAs such as n-butyl in mesh fixation on the abdominal wall by showing results similar to those of sutures, with good integration of the prosthesis, normal inflammatory reactions, generally good local tolerance, and optimal biocompatibility, affirming a positive cost/benefit analysis compared with other surgical techniques.

All these positive results in the literature supporting chemical tissue adhesives demonstrate the relevance that CAs are acquiring in current, modern surgical practice. Although further studies need to be conducted to evaluate the long-term performance of these novel tissue adhesives in mesh fixation in hernia repair, these compounds are already very attractive alternatives for this type of surgical procedure.

## Conclusions

In summary, our findings indicate the following:

*In vitro*, the longest side-chain CA (OCA) exhibited mild formaldehyde release and was the most cytotoxic during polymerization; however, it was the least cytotoxic once cured.

In the *in vivo* preclinical study, all CA-based tissue adhesives promoted proper mesh fixation, showing good tissue integration and effective short term biocompatibility; the slightest seroma formation and the lowest macrophage response resulted from the longest side-chain CA (OCA).

These results demonstrate the promise of these materials for replacing traditional suturing techniques in hernia repair.
